# The NLRP3 inflammasome in ischemic stroke

**DOI:** 10.3389/fstro.2024.1382379

**Published:** 2024-06-14

**Authors:** Sepiso K. Masenga, Annet Kirabo

**Affiliations:** ^1^HAND Research Group, School of Medicine and Health Sciences, Mulungushi University, Livingstone, Zambia; ^2^Department of Medicine, Vanderbilt University Medical Center, Nashville, TN, United States; ^3^Vanderbilt Center for Immunobiology, Nashville, TN, United States; ^4^Vanderbilt Institute for Infection, Immunology, and Inflammation, Nashville, TN, United States; ^5^Vanderbilt Institute for Global Health, Nashville, TN, United States

**Keywords:** NLRP3, inflammasome, ischemic stroke, genetic susceptibility, cardiovascular disease, brain, caspase-1

## Abstract

Ischemic stroke is a more common type of stroke and a leading cause of physical disability, cognitive decline, and death worldwide. Events occurring after an ischemic stroke episode determine the severity and outcomes. The NLR family pyrin domain containing 3 (NLRP3) inflammasome has emerged as a major contributor to the pathogenesis of ischemic stroke. Understanding its role in propagating ischemic injury is cardinal for therapeutic interventional research. In this review we summarize the current understanding of the underlying role of the NLRP3 inflammasome as well as highlight the current strides made in targeting the inflammasome as a modality to attenuate the effects of ischemic injury on brain tissue after a stroke event. We found that ischemic stroke initiates a cascade of complex intracellular processes beginning with oxidative stress that activates the nuclear factor kappa-light-chain-enhancer of activated B cells (NF-κB) consequentially activating the NLRP3 inflammasome. The NLRP3 inflammasome initiates inflammatory responses that exacerbate ischemic stroke. We have also briefly summarized the role of genetic susceptibility in stroke and its potential usage in clinical settings. Briefly, genetic mutations encoding the NLRP3 inflammasome are linked to stroke prognosis. A combination of advanced genetic testing and risk stratification based on sociodemographic, dietary, and lifestyle factors is encouraged for stroke prevention. IL-1β and IL-18 antagonists have been shown to inhibit the NLRP3 inflammasome consequently attenuating the adverse effects of ischemic stroke.

## Graphical Abstract

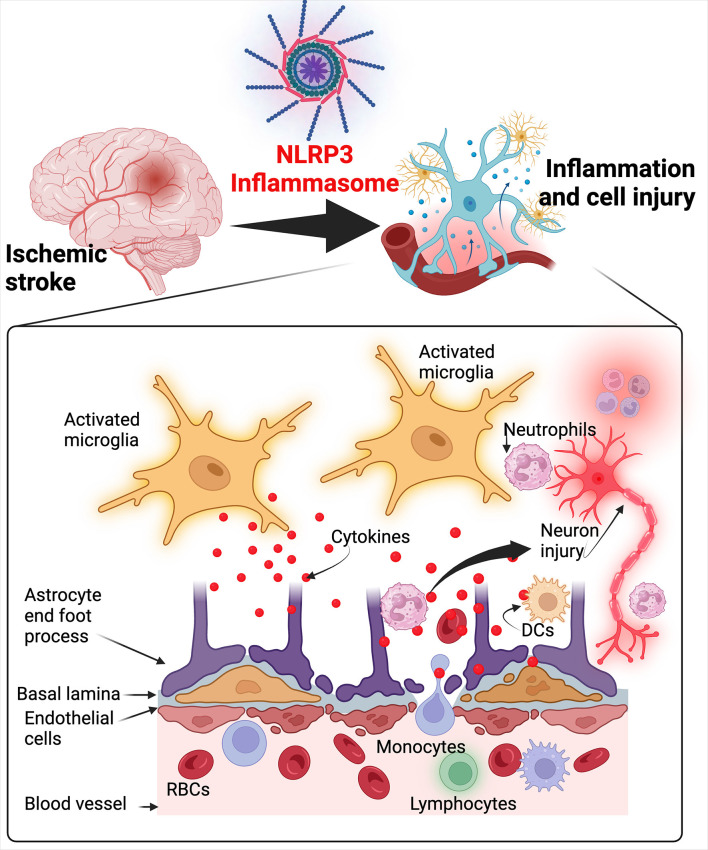

## 1 Introduction

Stroke is the second leading cause of death and disability worldwide accounting for more than 5.5 million deaths each year with the bulk of the global stroke burden in low-and-middle-income (LMICs) (Lopez et al., 2006; Feigin et al., 2022). There are two major categories of stroke, ischemic and hemorrhagic. Ischemic stroke is characterized by occlusion of a blood vessel and supply to a part of the brain leading to loss of function (Donkor, 2018). Haemorrhagic stroke is characterized by rupture of a blood vessel in the brain (Donkor, 2018). In some cases, a transient ischemic attack (TIA) can occur and is characterized by blockage of blood flow to the brain occurring only for a short time (<5 min). Of the two major categories of shock, Ischemic shock is more prevalent (Johnston et al., 2009) and is projected to increase by 2030 (Pu et al., 2023).

The common risk factors for both ischemic and haemorrhagic stroke include but not limited to hypertension, cardiovascular diseases, diabetes mellitus, hypercholesterolemia, atrial fibrillation, smoking, alcohol consumption, old age, female sex, use of oral contraceptives, hyperuricemia, chronic bronchitis, periodontal disease, bacterial and viral infections, and poor dietary lifestyle including high salt intake, race and ethnicity, family history and genetic factors (Connor et al., 2007; Owolabi et al., 2009; Palm et al., 2009; O'Donnell et al., 2010; Li et al., 2012; Pu et al., 2023).

The underlying pathophysiology of ischemic stroke stems from the fact that occlusion of blood supply affects brain cell function resulting in hypoxia and hypoglycemia of the affected tissue leading to an infarction (Doyle et al., 2008; Donkor, 2018). In hemorrhagic stroke the hematoma causes an increase in intracranial pressure and the metabolic waste from the ruptured vessel including the formed clots and a complex interaction involving edema, inflammation and oxidative stress contribute to tissue injury and death of brain cells (Magid-Bernstein et al., 2022). In ischemic stroke, the NLR family pyrin domain containing 3 (NLRP3) inflammasome has been reported to play a critical role in contributing to the ischemic injury following blood vessel occlusion by increasing oxidative stress and the inflammatory response (Chen et al., 2019). However, the underlying mechanisms are not well elaborated. The risk and severity of Ischemic stroke and the role of the NLRP3 inflammasome are to a degree dependent on genetic polymorphisms in the genes encoding the NLRP3 inflammasome as well as specific single gene disorders associated primarily with the development of stroke, or polymorphisms resulting in disease conditions where stroke is a common manifestation. Understanding this genetic component is critical for specific therapeutic interventional modalities to prevent and reduce the risk of stroke. Moreover, identification of mutations encoding the NLRP3 inflammasome is important in understanding its role, phenotypic expression, and susceptibility to stroke.

In this review, we discuss in detail, the role of the NLRP3 inflammasome in the pathogenesis of ischemic stroke and later provide a brief current understanding of genetic susceptibility to ischemic stroke.

## 2 Pathophysiology and mechanisms of ischemic stroke

The main pathological mechanisms associated with cell injury and death in cerebral ischemic stroke are atherosclerosis, endothelial dysfunction, and neuroinflammation (Tuttolomondo et al., 2020). These mechanisms are mediated by several cellular pathological processes such as oxidative stress, inflammation, ATP depletion, mitochondrial damage, influx of Ca^2+^, defects in membrane permeability, and damage to DNA and intracellular proteins and organelles, all resulting in reversible and irreversible endothelial, glia, and neuronal cell injury depending on the nature and severity of the ischemia (Anrather and Iadecola, 2016).

**Figure 1:**
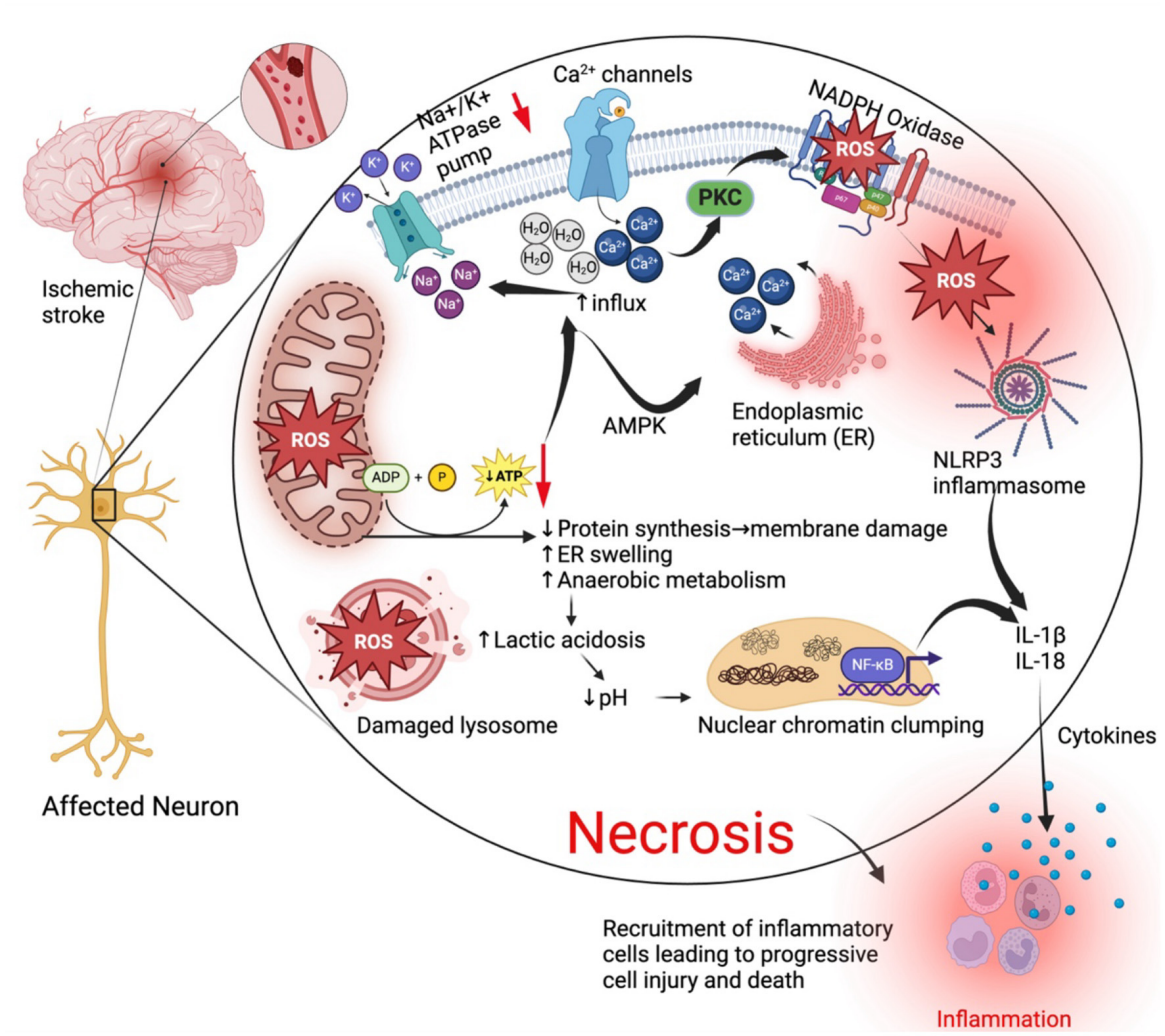
General cell death mechanisms in ischemic stroke. During an ischemic stroke when blood supply is cut off to an area of the brain, affected neurons go through a series of pathological changes leading to cell death by necrosis and elicit inflammatory responses that further exacerbate and accelerate injury. Lack of oxygen reduces oxidative phosphorylation (ATP production) in the mitochondria and lead to mitochondria dysfunction and oxidative stress that contributes to production of reactive oxygen species (ROS). Reduced ATP production leads to reduced protein synthesis, swelling of the endoplasmic reticulum and malfunction of the Na^+^/K^+^ ATPase pump. Reduced activity of the Na^+^/K^+^ ATPase pump results in increased Na+ and water retention in the cell causing swelling of intracellular organelles. ATP reduction or depletion leads to influx of calcium from outside the cell and its release from the ER mediated by AMP-activated kinase. Ca^2+^ activates protein kinase c (PKC). PKC activates the enzyme NADPH oxidase which increases activity and production of ROS. ROS activate the nuclear factor kappa-light-chain-enhancer of activated B cells (NF-κB) which activates the NLRP3 inflammasome and both contribute to the production of interleukin 1 beta (IL-1β) and IL-18 resulting in inflammatory responses. Cell death arising from these necrotic neurons also elicit an inflammatory response that recruits inflammatory cells, exacerbating cell injury. Created with BioRender.com.

Of the two main prominent cell types of the brain, glial cells and the neurons (Siletti et al., 2023), neurons are more susceptible to ischemic injury from cerebral ischemia (Won et al., 2022). Occlusion of a blood vessel in the brain leads to lack of oxygen and glucose required for major metabolic pathways such as the mitochondria oxidative phosphorylation that provide adenosine triphosphates (ATPs) needed by the cell (Ham and Raju, 2017; Huang J. et al., 2023). Reduced oxidative phosphorylation results in ATP depletion that has multiple pathological consequences for the brain cells (Guo et al., 2013; Clemente-Suárez et al., 2023). Lack of ATP causes a disruption of the Na^+^/K^+^ ATPase pump that leads to increased influx of Ca^2+^, water and sodium and increased efflux of K+ resulting in endoplasmic reticulum swelling, cellular swelling and loss of microvilli blebs (Gusarova et al., 2011; Miller and Zachary, 2017). Influx of Ca^2+^ via Ca^2+^ release-activated Ca^2+^ (CRAC) channels and release of Ca^2+^ from the endoplasmic reticulum during hypoxia is mainly mediated by the activation of AMP-activated kinase α1 (AMPK-α1) through Ca^2+^/calmodulin-dependent kinase β (CaMKKβ) and redistribution of the stromal interaction molecule 1 (STIM1) to the plasma membrane- endoplasmic reticulum junctions (Gusarova et al., 2011). Cellular swelling leads to cytotoxic edema and cell death (Brouns and De Deyn, 2009) (Figure 1). Suffice to say that these pathological events are common to all cells during ischemia, neurons and almost all brain cells are more susceptible to cell death and adverse outcomes following an ischemic stroke episode.

The metabolic consequences of reduced ATP production results in increased anaerobic metabolism leading to increased lactic acid and clumping of nuclear chromatin (Miller and Zachary, 2017). Reduction in ATP also leads to detachment of ribosomes from the rough endoplasmic reticulum, consequentially leading to reduced protein synthesis, phospholipid synthesis and reacylation, membrane damage causing leakage of cellular contents that elicit an inflammatory response (Miller and Zachary, 2017; Almanza et al., 2019; Perkins and Allan, 2021). Ischemia is also associated with increased production of reactive oxygen species (ROS) due to deranged metabolic systems, and this in turn results in an oxidative burst by injured cells that are trying to survive (Olmez and Ozyurt, 2012; Rodrigo et al., 2013; Elsayed et al., 2020). ROS peroxidize intracellular proteins, lipids, and cellular organelles such as lysosomes that leak their digestive contents to the outside resulting in Ischemic cell death by necrosis (Alu et al., 2020; Ammendolia et al., 2021; He et al., 2023).

Several pro-inflammatory mediators such as IL1β, IL1α, TNF-α, leukotrienes, and chemokines including chemokine (C-X-C motif) ligand 4 (CXCL4), chemokine (C-C motif) ligand 5 (CCL5), CXCL7, CXCL8, chemokine (C-X3-C motif) ligand 1 (CX3CL1), adhesion molecules, proteases, prostanoids, inducible nitric oxide synthase (iNOS), cyclooxygenase 2 (COX-2), are heightened in stroke suggesting their pathological role (Puleo et al., 2022). The inflammatory response in ischemic stroke is responsible for the secondary injury that develops in a delayed manner (Brouns and De Deyn, 2009). The first step associated with inflammation in ischemic stroke is the activation of microglia and endothelial cells (Stoll, 2002) which is followed by infiltration of innate cells such as neutrophils, macrophages, natural killer cells and T lymphocytes that release cytokines, ROS and cytotoxic compounds responsible for further injury to ischemic cells (Wang et al., 2007; Fu et al., 2015). When endothelial cells are activated, they increase the expression of adhesion molecules such as E-selectin, P-selectin, vascular cell adhesion protein 1 (VCAM-1) and intercellular adhesion molecule 1 (ICAM-1) that lead to leukocyte rolling, recruitment, and finally, diapedesis through the vascular wall (Wang et al., 1994; Huang et al., 2000). Neutrophils are also attracted to the infarcted area during reperfusion via monocyte chemo-attractant protein-1 (MCP-1) secreted by activated glial cells and neurons (Geng et al., 2022). Reperfusion also activates a wide variety of cells like macrophages, neutrophils and natural killer cells that secret more pro-inflammatory cytokines and matrix metalloproteinases which disrupt the blood brain barrier exacerbating cerebral edema and activate iNOS and COX-2 which induce endothelial dysfunction leading to cell death (Yang et al., 2019; Geng et al., 2022; He et al., 2022).

The death of microglia and neuronal cells triggers an inflammatory response from surrounding immune cells via specific sensing mechanisms such as the inflammasome that will be discussed in more detail below.

## 3 NLRP3 inflammasome in ischemic stroke

### 3.1 NLRP3 inflammasome

**Figure 2:**
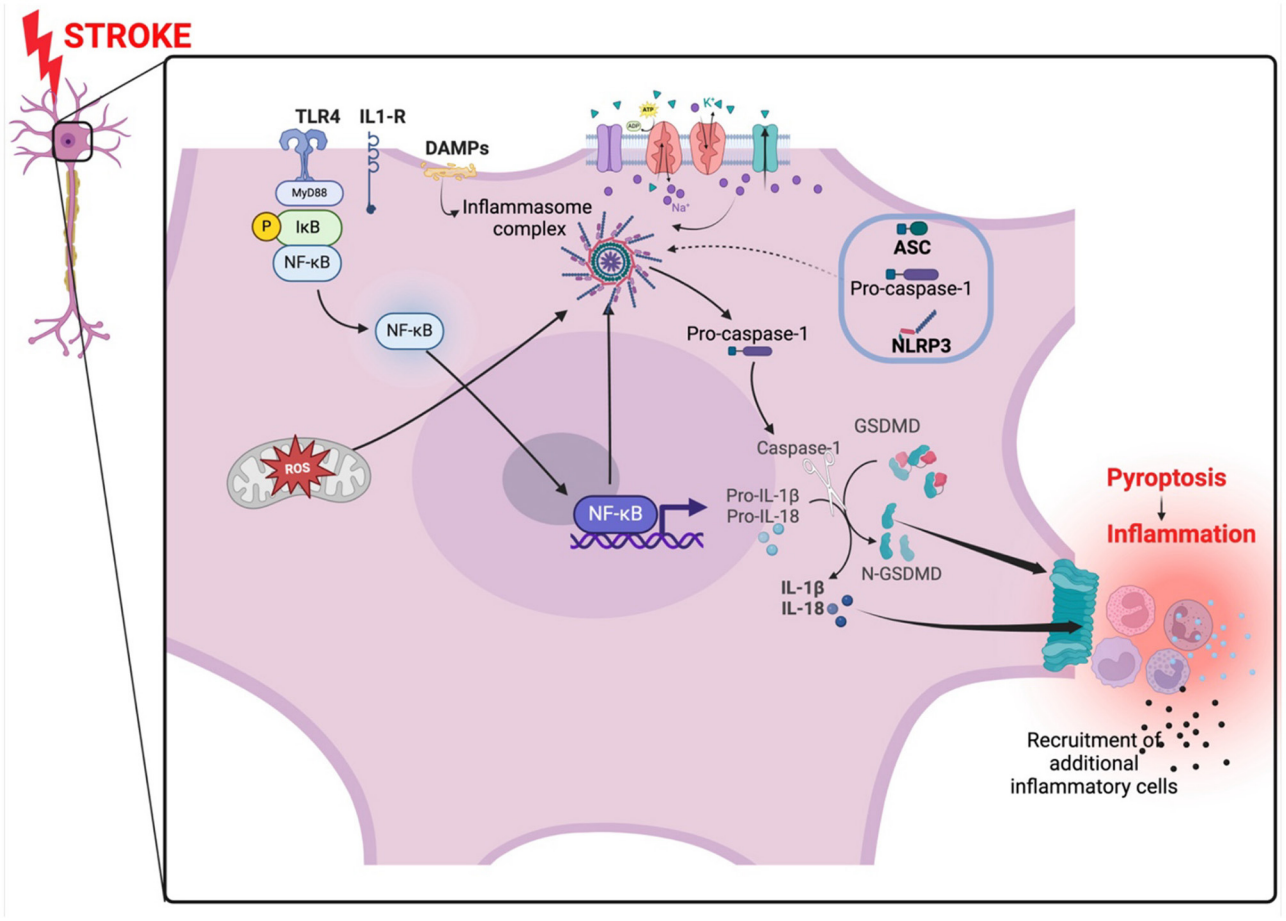
NLRP3 inflammasome activation in ischemic stroke. The inflammasome is activated by oxidant stress created by the occlusion. The oxidant stress from the mitochondria arises from a lack of oxygen and nutrients. An abnormal influx and inflow of ions from disrupted ion gated channels and NF-κB also activated the inflammasome complex which in turn activate caspase-1 leading to the activation of IL-1β and il-18. Gasdermin forms pores on the membrane promoting pyroptosis and subsequently, inflammation. The NF-κB pathway is activated via MyD88 signaling. TLR4, toll-like receptor 4; NLRP3, NLR family pyrin domain containing 3; ASC, apoptosis-associated speck-like protein; DAMPs, damage-associated molecular patterns; GSDMD, gasdermin D; NF-κB, nuclear factor kappa-light-chain-enhancer of activated B cells; MyD88, Myeloid differentiation primary response 88. Created with BioRender.com.

The NLRP3 Inflammasome is a multimeric protein complex expressed mainly by innate immune cells and a variety of other cells such as microglia, lymphocytes, epithelial cells, osteoblasts, and neurons (Rada et al., 2014; Zahid et al., 2019). The NLRP3 Inflammasome contains three critical domains that regulate inflammatory signaling (Lebreton et al., 2018; Blevins et al., 2022). The three domains include the NLRP3 protein, the apoptosis-associated speck-like protein (ASC) which is an adaptor protein, and a procaspase-1, an effector enzyme (Zoete et al., 2014; Mamantopoulos et al., 2017). The NLRP3 protein is made up of a central nucleotide-binding and oligomerization domain (NOD or NACHT), amino-terminal pyrin domain (PYD) responsible for interacting with ASC and initiating inflammasome assembly, and a C-terminal leucine-rich repeat (LRR) domain (Franchi et al., 2009; Vajjhala et al., 2012). NLRP3 protein is a cytosolic pattern recognition receptor (PRR) that functions as a sensor protein which responds to endogenous stress-generated pathogen-associated molecular patterns (PAMPs) or damage-associated molecular patterns (DAMPs) (Kelley et al., 2019). When stimulated the sensor protein NLRP3 forms a supramolecular molecular complex referred to as “inflammasome” which then activates pro-caspase-1 (Fernandes-Alnemri et al., 2007). Pro-caspase-1 is activated to caspase-1 which in turn catalyzes the cleavage of pro-interleukin-1β (pro-IL-1β) and pro-IL-18 into active IL-1β and IL-18 (Manji et al., 2002). Caspase-1 also cleaves gasdermin D (GSDMD) which induces pyroptosis by forming pores in the plasma membrane of cells releasing DAMPs and eliciting an inflammatory response (Shi et al., 2015). IL-1β and IL-18 induce vasodilation, interferon-gamma (IFN-γ) production and recruitment of immune cells to injured tissues (Dinarello, 2009) (Figure 2). It is important to also highlight that absent-in-melanoma 2 (AIM2), a cytoplasmic sensor, can also interact with the PYD domain and when activated can process and contribute to the secretion of IL-1β and IL-18 (Kumari et al., 2020). AIM2 is also an inflammasome that particularly senses DNA (damaged from cell injury or health cell) in the cytosol and trigger the secretion of IL-1β and IL-18 (Kumari et al., 2020).

PRRs that are known to form inflammasomes include leucine-rich repeat (LRR)-containing proteins (NLR) family members NLRP1, NLRP2, NLRP3, NLRC4, NLRP6, NLRP7, NLRP12, IFI16, pyrin, AIM2, and nucleotide-binding oligomerization domain (NOD) (Elinav et al., 2011; Vladimer et al., 2012; Minkiewicz et al., 2013; Lamkanfi and Dixit, 2014; Sharma and Kanneganti, 2016). Among the variety of inflammasomes known to man, the NLRP3 inflammasome was not the first to be discovered but is the most studied and therefore a focus of this review (Bulté et al., 2023; Xu and Núñez, 2023). Although the NLRP3 inflammasome is central to immune responses against infectious agents, it has been reported to be activated and contribute to conditions that do not necessarily involve infections (Guo et al., 2015).

Generally, the activation of the NLRP3 inflammasome in cell injury requires two complementary signals; the first signal is mediated by nuclear factor kappa-light-chain-enhancer of activated B cells (NF-κB) and mitogen-activated protein kinase (MAPK) signaling pathways that serve to upregulate proteins of the NLRP3 inflammasome complex and the precursors of IL-1β and IL-18, and the second signal serves to assemble the NLRP3 inflammasome complex via activation of NLRP3 and ASC phosphorylation (Abais et al., 2015).

#### 3.1.1 Activators of the NLRP3 inflammasome

The NLRP3 inflammasome can be activated by a wide range of stimuli including ROS, ionic influx, mitochondrial and lysosomal damage among others (Hornung et al., 2008; Kelley et al., 2019). Events leading to potassium (K^+^) efflux and depletion of K^+^ have been reported to activate the NLRP3 inflammasome and lead to the activation of Ca^2+^ independent phospholipase A2, resulting in IL-1β maturation and release from macrophages and monocytes (Walev et al., 2000; Muñoz-Planillo et al., 2013). NLRP3 inflammasome activation is associated with increases in cytosolic Ca^2+^ concentrations suggesting that Ca^2+^ is critical for NLRP3 inflammasome activation (Weber and Schilling, 2014). Multiple stimuli including high intracellular ATP, nigericin, phospholipase C activation via G-protein-coupled receptors (GPCRs) trigger Ca^2+^ mobilization from extracellular stores via plasma membrane Ca^2+^ channels, the lumen of the endoplasmic reticulum and lysosomes (Murakami et al., 2012; Weber and Schilling, 2014). Na+ influx and Cl– efflux have also been implicated in NLRP3 inflammasome activation as the blockade of Na+ influx by gramicidin, K+-free medium, or nigericin and inhibition of Cl– efflux by 5-nitro-2-(3-phenylpropylamino) benzoic acid, 4,40-diisothiocyano-2,20-stilbenedisulfonic acid, mefenamic acid, flufenamic acid, and indanyloxyacetic acid 94, inhibits NLRP3 inflammasome activation and IL-1β secretion (Verhoef et al., 2005; Compan et al., 2012; Muñoz-Planillo et al., 2013; Daniels et al., 2016; Tang et al., 2017). ROS from NADPH oxidase, mitochondrial, lysosomes and other cellular organelles have also been reported to activate the NLRP3 inflammasome (Bauernfeind et al., 2011; Nakahira et al., 2011; Zhou et al., 2011).

#### 3.1.2 The NLRP3 inflammasome in ischemic stroke

The NLRP3 inflammasome is well expressed in the brain and spinal cord and during stroke, the member proteins of the NLRP3 inflammasome including ASC, caspase-1, IL-1β and IL-18 were upregulated *in vitro* and *in vivo* (Iadecola and Anrather, 2011; Yang-Wei Fann et al., 2013; Creagh, 2014; Zhao et al., 2021).

Ischemic stroke has been reported to induce activation of the NF-κB and MAPK signaling pathways in ischemic neurons in the brain promoting NLRP3 Inflammasome activation and subsequent neuronal cell death and brain injury (Fann et al., 2018). The activation of the NF-κB occurs following the priming signal where cellular debris and DAMPs from damaged parenchyma in ischemic stroke are recognized by PRRs (Kelley et al., 2019). PRRs then serve to activate NF-κB and NLRP3 inflammasome eliciting the transcription and translation of IL-18 and IL-1β levels (Kelley et al., 2019; Kumar, 2019). Two independent adaptor proteins are implicated in the NF-κB signaling pathway which is associated with NLRP3 inflammasome activation and induction of pro-inflammatory cytokines IL-18 and IL-1β. The Myeloid differentiation primary response 88 (MyD88) and TIR-domain-containing adapter-inducing interferon-β (TRIF) are two adaptor proteins that can initiate NLRP3 inflammasome activation separately via TLRs ligand interactions such as TLR4 (Duran et al., 2016; Zamyatina and Heine, 2020; Kim et al., 2023). Through the NF-κB-MyD88 dependent pathway, activation of TLR4 leads to the binding of MyD88 to the cytoplasmic portion of the TLR4 initiating the recruitment of interleukin-1 receptor-associated kinase 4 (IRAK-4) and IRAK-1 to form a complex (Puleo et al., 2022). IRAK-4 activates IRAK-1 by phosphorylation dependent mechanisms leading to its attachment to the tumor necrosis factor receptor associated factor 6 (TRAF6) and forming a quadruplet complex called myddosome (Puleo et al., 2022). IRAK-1 and TRAF-6 then activate IκB kinase-β (IKK -β) which leads to the degradation of IκB by means of phosphorylation causing the release and migration of NF-κB into the nucleus to elicit transcription and translation of pro-IL-1β and NLRP3 expression in priming the NLRP3-inflammasome for activation (Boaru et al., 2015; Zhong et al., 2016; Liu et al., 2022) (Figure 2). In the TRIF-dependent pathway, TRIF binds to the TLR4 together with tripartite interaction motif (TRIM) and activate trafficking kinesin-binding protein 1 (TAK-1) which then attaches to TRAF6 and initiates NLRP3-inflammasome activation via NF-κB in a similar fashion as the MyD88-dependent pathway (Kumar, 2019). The priming of NLRP3-inflammasome for activation is preceded by phosphorylation of ASC, and the deubiquitination and activation of NLRP3 which is mediated by a JAMM domain-containing Zn^2+^ metalloprotease called BRCC36 (Py et al., 2013). ASC then assembles with NLRP3 and procaspase-1 forming the NLRP3 inflammasome complex. Procaspase-1 is cleaved to caspase-1 which converts proinflammatory IL-1β and IL-18 into active IL-1β and IL-18 that promote neuroinflammatory damage in ischemic stroke (Liu et al., 2021).

The group by Fann et al. (2018) demonstrated that reducing activation of the NF-κB and MAPK pathways using intravenous immunoglobulins in ischemic stroke ameliorated cell death and injury by reducing the expression and activation of the NLRP1 and NLRP3 inflammasomes, and increasing expression of anti-apoptotic proteins, Bcl-2 and Bcl-xL in primary cortical neurons and/or cerebral tissue. Further, restoration of blood supply which is a key aspect in the treatment of stroke, results in cerebral ischemic-reperfusion injury mainly from the increased production of ischemia-induced production of ROS and activation of NLRP3 inflammasome that exacerbates injury and inflammation induced injury causing more brain cell damage and dysfunction, and brain edema (Wang et al., 2007; Minutoli et al., 2016).

#### 3.1.3 Mechanism of injury associated with NLRP3 inflammasome activation

The NLRP3 inflammasome contributes to brain cell injury through various mechanisms. Franke et al. (2021) demonstrated that the NLRP3 inflammasome is mainly expressed within ischemic neurons and drives inflammation in ischemia/reperfusion injury following transient middle cerebral artery occlusion in mice. In atherosclerosis, which is an underlying process that mediates ischemic stroke, NLRP3 is activated inside foam cells by ROS released by ruptured lysosomes leading to increased release of inflammatory cytokines such as IL-18 and IL-1β (Hua et al., 2013). IL-18 promotes necrosis of vascular smooth muscle cells releasing tissue metalloproteinases that destabilize plaque stability, increasing atherothrombotic and occlusion tendency (Zheng et al., 2013). Oxidized LDL that forms fatty streaks activates toll-like receptor type 12 or 4 (TLR-12/TLR-4) signaling that in turn activate downstream signal transduction molecules leading to the induction of myeloid differentiation primary response gene 88 (MyD88) which in turn leads to expression of NF-κB via interferon TIR-domain-containing adapter-inducing interferon-β (TRIF)-mediated mechanisms (Segovia et al., 2012; Tarallo et al., 2012). As discussed earlier, NF-κB promotes NLRP3 inflammasome activation and activation of pro- IL-1β via caspase-1 dependent mechanism to promote inflammation that further exacerbates ischemic injury (Segovia et al., 2012). IL-1β is a pleiotropic important cytokine involved in the activation of several proinflammatory signaling pathways in peripheral tissue including microglia and astrocytes in the brain during ischemic stroke (Mendiola and Cardona, 2018). IL-1β contributes to toxic cerebral edema and inflammation by disrupting the blood brain barrier and facilitating infiltration of immune cells in the central nervous system (Jagadapillai et al., 2022). In animal models, messenger ribonucleic acid (mRNA) levels of IL-1β are increased by 30 min post-cerebral ischemia and after a few hours IL-1β protein expression levels are also increased suggesting the pathological role of IL-1β in ischemic stroke (Buttini et al., 1994; Davies et al., 1999; Caso et al., 2007). This is evidenced by increased brain injury following administration of IL-1β and reduced infarct volume in IL-1β-deficient mice and mice administered with IL-1 receptor antagonist (IL-1ra) (Yamasaki et al., 1995; Boutin et al., 2001; Pradillo et al., 2012; Murray et al., 2015; Flygt et al., 2018; Ozen et al., 2020). One proposed mechanism mediating ischemic brain injury by IL-1 which is secreted by neutrophils is that IL-1 induces IL-17A in γδ T Cells and CXCL1 in Astrocytes to promote and amplify the post-stroke inflammatory response (Schädlich et al., 2022). CXCL1 is a specific chemokine attractant of neutrophils and IL-17A synergizes with TNF to enhance the expression of CXCL1 in astrocytes (Schädlich et al., 2022). Apart from increasing expression levels of CXCL1, systemic administration of IL-1β also increases macrophage inflammatory protein-2 (MIP-2) further enhancing neutrophil recruitment and infiltration and production of neutrophil-derived matrix metalloproteinase-9 (McColl et al., 2007, 2008).

Apart from IL-1β, activated microglia and astrocytes also secret TNF-α, transforming growth factor-β (TGF-β), interferon-β (IFN-β), MCP-1, stromal cell-derived factor 1 (SDF-1), macrophage inflammatory protein-1α (MIP-1α), and matrix metalloproteinases that disrupt the basal lamina of the blood brain barrier and gap junctions between endothelial cells increasing intravascular permeability, cell adhesion and diapedesis of recruited cells which then infiltrate the brain and cause more neuronal injury (Yang et al., 2019).

The early pathological processes resulting in inflammasome activation and induction of inflammation in ischemic stroke induces activation and infiltration of vascular smooth muscle cells, neutrophils, macrophages, lymphocytes, into the ischemic area, worsening tissue injury and inducing fibrotic changes (McDonald et al., 2010; Artlett, 2012).

## 4 Therapeutic advances targeting NLRP3 inflammasome in ischemic stroke

Although development of drugs to target the NLRP3 inflammasome are underway, several therapeutic modalities do exist that can potentially be used to ameliorate ischemic injury in stroke (Feng et al., 2020; Franke et al., 2021). Animal studies have demonstrated the protective effect against neurological deterioration and infarct volume resulting from inhibition of the NLRP3 inflammasome after ischemic stroke (Deroide et al., 2013; Yang et al., 2014). Drugs that inhibit IL-1β such as the anti-IL-1β antibody canakinumab or antagonists of IL-1 receptor such as anakinra hormone or NLRP3 inhibitor such as atorvastatin are currently being studied and are known to reduce IL-1β levels by inhibiting activation of the NLRP3 inflammasome (McGonagle et al., 2007; Carné, 2011; Ridker et al., 2011, 2012; Satoh et al., 2014). Other drugs such as ritonavir, an inhibitor of caspase-1 and P2X7 receptor antagonists also reduce IL-1β and IL-18 levels (Stokes et al., 2006; Arulkumaran et al., 2011; López-Castejón and Pelegrín, 2012). Other drugs being studied that have potential to inhibit inflammasome activation and ameliorate ischemic stroke tissue injury include minocycline administrated 1 hour following reperfusion (Lu et al., 2016), IMM-H004 (Ai et al., 2019), progesterone and steroids 17b-estradiol (Lammerding et al., 2015; Zhang et al., 2017), Stachybotrys microspora triphenyl phenol-7 (SMTP-7) (Huang et al., 2018), MCC950 (Ismael et al., 2018), and other agents which have been described elsewhere (Feng et al., 2020).

However, due to the acute nature of the pathological changes occurring in ischemic stroke, timing, and dosage of these drugs remain unclear, requiring more studies (Gao et al., 2017).

Minimizing the inflammatory response after stroke is another modality of ameliorating the secondary adverse effects mediated by inflammatory cells. Use of anti-ICAM-1 antibody in transient focal cerebral ischemia model reduced cellular infiltrates into the brain leading to reduced ischemic brain damage (Zhang et al., 1994). However, clinical trials using anti-ICAM-1 antibody enlimomab was associated with further damage and adverse effects in human clinical trials (Furuya et al., 2001; Enlimomab Acute Stroke Trial Investigators, 2021). Other drugs such as IL-1RA antagonist, broad-spectrum tetracycline antibiotic minocycline, fingolimod an agonist of sphingosine-1-phosphate receptor, celecoxib (a selective inhibitor of cyclooxygenase 2), etanercept a TNFα inhibitor, and peroxisome proliferator-activated receptor gamma (PPAR-γ) agonists are being explored (Chu et al., 2004; Luo et al., 2006; Lampl et al., 2007; Fu et al., 2014; Lu et al., 2016; Lee et al., 2017; Duris et al., 2018).

Virtually all brain cells including neurons, astrocytes and microglia of both humans and rodents express the NLRP3 inflammasome but with variations in intensity and mechanisms (Chiarini et al., 2020; Pike et al., 2021). While the NLRP3 inflammasome is also expressed in neurons and astrocytes, the human microglia seems to predominate the expression (Lech et al., 2010; de Rivero Vaccari et al., 2014; Nyúl-Tóth et al., 2017; Pike et al., 2021; Sandhu and Kulka, 2021). As already discussed, activation of the NLRP3 inflammasome in human and rodent microglia is associated with inflammatory responses that exacerbate pathogenesis of injury including ischemic stroke injury. Pharmacological interventions that inhibit NLRP3 inflammasome activation appears to decrease this inflammatory response and attenuates further injury (de Rivero Vaccari et al., 2014). Although several therapeutics targeting inflammasomes are being investigated, there is scarcity of data to allow a comparison of these interventions with others used to treat ischemic stroke. Future studies to investigate this aspect are therefore warrantable.

## 5 Genetic susceptibility of ischemic stroke

### 5.1 NLRP3 inflammasome genetic susceptibility

Genetic mutations involving the NLRP3 inflammasome gene associated with stroke are still being studied and still new in the field. However, in a recent study by Kumar et al. (2022) conducted in an Indian population, they found that a susceptibility putative haplotype GTGTA within NLRP3 gene conferred a double risk of ischemic stroke in carriers and potentiated increased levels of c-reactive protein (CRP) and IL-1β in a dose-dependent manner.

In another study from China by Lv et al. (2020), they found that men who were carrying both caspase recruitment domain family member 8 (CARD8) rs2043211 AT and NLRP3 rs10754558 CG were seven times more likely to develop ischemic stroke compared to women. In another study among the Chinese Han population, the NLRP3 gene polymorphism rs4612666 increased susceptibility of large artery atherosclerosis (LAA) ischemic stroke by influencing plaque vulnerability (Cheng et al., 2018).

### 5.2 Genetic polymorphisms and mechanisms related to stroke risk in general

Several gene polymorphisms associated with susceptibility to ischemic stroke have been identified including the CYP11B1 gene (rs5283) and CYP4F2 gene (rs2108622, rs3093106, and rs3093105) in Chinese Han population (Liu and Duan, 2022; Huang K. et al., 2023). In addition, the diabetes risk variants (rs7578326 and rs12779790) and the susceptibility allele for myocardial infarction, the CDKN2A/B locus (rs2383207, 9p21) in the Religious Orders Study (ROS) and the Rush Memory and Aging Project (MAP) from the USA were also identified to increase the risk for ischemic stroke (Chou et al., 2013). Several other common variants in polygenic diseases are described elsewhere (Terni et al., 2014; Ekkert et al., 2022).

Genetic risk for ischemic stroke occurs through various mechanisms including single gene disorders associated primarily with the development of stroke and genetic mutations resulting in disorders where stroke is one of the manifestations. In addition, common genetic variants associated with the risk for stroke including polymorphisms that are associated with increasing the risk for those diseases that also increase the risk for stroke play a critical role in increasing susceptibility.

Small vessel diseases such as Cerebral Autosomal Dominant Arteriopathy with Subcortical Infarcts and Leukoencephalopathy (CADASIL) and Cerebral Autosomal Recessive Arteriopathy with Subcortical Infarcts and Leukoencephalopathy (CARASIL) are autosomal dominant and recessive diseases, respectively, associated with ischemic stroke and leukoencephalopathy (Boehme et al., 2017). CADASIL is associated with single gene mutation of the Notch3 gene encoding a transmembrane surface protein mainly expressed in vascular cells and plays a major role in cellular signaling pathways (Locatelli et al., 2020). CARASIL is associated with mutations in the high-temperature requirement-A (HtrA) serine peptidase-1 gene encoding an enzyme that plays a major role in defense against cellular stresses (Uemura et al., 2020; Grigaite et al., 2021).

The mutations in both Notch3 and HtrA serine peptidase-1 result in susceptibility to ischemia. Other single gene disorders that are associated with stroke include familial amyloid angiopathy and collagen 4 (COL4A1) mutations (Tan et al., 2019; Chojdak-Łukasiewicz et al., 2021). The underlying pathology associated with these diseases include rupture of small cortical vessels due to defects in collagen and deposition of amyloid plaques. Genetic disorders where ischemic stroke is a manifestation include Smooth Muscle Alpha-Actin (ACTA2) mutation associated disorders, sickle cell disease, Ehlers-Danlos Type 4, Marfan syndrome, fabry disease and Mitochondrial encephalopathy with lactic acid and stroke-like episodes (MELAS) (Boehme et al., 2017).

Heritability of ischemic stroke is lower for small vessel disease and higher and similar for cardioembolic and large vessel diseases (Bevan et al., 2012). Generally, family history and monozygotic twins have more than a 30% higher risk for ischemic stroke (Flossmann et al., 2004).

Additional modifiable and non-modifiable risk factors that may exacerbate stroke heritability and trigger stroke include age, female sex with parental history of stroke, (Schulz et al., 2004; Touzé and Rothwell, 2008), pollution, disorders where inflammation is the hallmark of pathogenesis, infectious diseases, cardiac atrial disorders, hypertension, diabetes, physical inactivity, hyperlipidemia, diet, alcohol consumption, apolipoprotein B to A1, race/ethnicity and smoking (Boehme et al., 2017).

Relating to age and sex, women have similar and sometimes higher risk for stroke at younger ages but during older ages, men have a slightly higher risk compared to women (Kapral et al., 2005). Regarding race and ethnicity, it is well established that the risk for stroke and stroke-related deaths is double in African Americans compared to their white counterparts (Kissela et al., 2004; Morgenstern et al., 2004; Zahuranec et al., 2006; Cruz-Flores et al., 2011; Ashley and Berry, 2020).

### 5.3 Clinical application

Common methods employed to discover genetic causes of ischemic stroke include candidate gene approaches, twin and family history studies, genome-wide association studies and next generation sequencing (Ekkert et al., 2022). Genetic susceptibility to ischemic stroke is more relevant when employing multilocus genetic risk scores and polygenic risk scores (polyGRS) because of the multifactorial etiological nature of ischemic stroke. However, due to the polygenic nature of ischemic stroke, the latter scoring technique also inferred from genome-wide association (GWA) studies but combining different subtypes related to susceptibilities to cardiometabolic and CVD risk such as arteriosclerosis, hypertension, hyperlipidemia is a better technique in estimating their cumulative contribution to stroke (Hachiya et al., 2017; Ekkert et al., 2022). A study by Hachiya et al. (2017) used this technique and found that use of polyGRS combined with a non-genetic risk model strengthened the predictive ability of ischemic stroke.

Thus, this method taken together with non-genetic risk factors for stroke is an emerging revolutionary concept that will soon gain ground in clinical practice. For now, this field is still growing with more studies required to advance the predictive technology of ischemic stroke in clinical practice to prevent cerebrovascular accidents and associated adverse outcomes.

## 6 Future perspectives

Ischemic stroke accounts for most CVD deaths and remains an important area of constant study. The underlying role of the inflammasome in ischemic stroke and therapeutic targets is an ongoing field of study. Future research must be more focused on targeting signaling pathways involved in the activation of the NLRP3 Inflammasome to ameliorate the severity of brain injury due to ischemic stroke. However, more studies are required that will focus on preventive therapy and use of polyGRS combined with a non-genetic risk factor stratification to predict ischemic stroke more accurately and thus discover ways to delay or prevent ischemic stroke.

Clinical studies that are focused on identifying biomarkers of ischemic stroke by interrogating the components of the NLRP3 inflammasome are also warrantable. As reported previously, inflammasome proteins such as caspase-1, ASC in serum, ASC and IL-18 in cerebral spinal fluid (CSF) are potential predictive biomarkers of traumatic brain injury (Kerr et al., 2018). ASC protein levels were higher in patients with poorer outcomes following traumatic brain injury suggesting that inflammasome proteins could be used in clinical settings as diagnostic and predictive biomarkers (Kerr et al., 2018). Although this study by Kerr et al. (2018) was not focused primarily on ischemic stroke and it is unclear whether the NLRP3 inflammasome proteins were the ones being measured, future clinical studies could explore this in ischemic stroke patients with varying severity in outcomes to determine the diagnostic and prognostic value of using NLRP3 inflammasome proteins in ischemic stroke.

For therapeutic interventions, use of NLRP3 inhibitors is a promising area for future research. The Canakinumab Anti-inflammatory Thrombosis Outcomes Study (CANTOS) is one of those successful trials demonstrating that that inhibition of NLRP3 inflammasome is beneficial in ameliorating the adverse outcomes of cardio-cerebrovascular events (Ridker et al., 2011, 2012; Everett et al., 2020). The CANTOS trial was primarily based on inhibiting the production of IL-1β. Future trials could therefore target additional proteins in the NLRP3 inflammasome activation cascade and compare efficacy and superiority with Canakinumab. In clinical settings, prompt diagnosis, prognosis, and treatment modalities are critical for stroke patient outcomes. More research should therefore explore this area. However, determining the risk of a stroke in an individual who has never had a stroke or an individual with a high risk for recurrent stroke is more critical and important in alleviating the burden of stroke globally, but it is currently a big challenge.

Genetic testing may be beneficial in determining persons who are at risk for ischemic stroke. Although integrating genetic testing in clinical practice is a challenge, current advances in this area are promising. The use of genome-wide association studies, next generation sequencing or simply genetic risk scores and extended polygenic risk scores to estimate the cumulative contribution of genetic factors for stroke development and then combining this information with clinical information and known existing risk factors for stroke may be a better method to prevent stroke. Although there is no established gold standard method to determine stroke risk in clinical settings, there are several biomarkers and genetic markers available for aiding decision making in clinical practice. A recent detailed description of how genetic testing can be incorporated in clinical practice has been elaborated by Ekkert et al. (2022).

## 7 Conclusions

Although ischemic stroke is fatal for the brain and associated with morbidity and mortality, understanding the intracellular and molecular pathogenesis is crucial for management modalities. Ischemic stroke activates several intracellular pathways that contribute to more injury and promote more death of brain tissue. Among the inflammasomes known to man, the most studied is the NLRP3 Inflammasome. After a stroke event, the activation of the NLRP3 Inflammasome via the MyD88-IRAK/TRAF- NF-κB pathway is associated with more inflammation and worsening of brain injury and outcomes. Targeting the NLRP3 inflammasome has potential to reduce the infarct volume after ischemic stroke and attenuate brain injury consequently reducing morbidity and disability. More clinical studies are therefore required that focus on targeting different aspects of the NLRP3 inflammasome signaling cascade to prevent recruitment and infiltration of brain tissue with activated inflammatory cells. Although the NLRP3 inflammasome is the most studied in disease, the underlying mechanisms related to the NLRP3 Inflammasome activation and exacerbation of ischemic injury are still elusive and complex, involving multiple intracellular signaling pathways.

The outcomes of stroke events are usually adverse, therefore preventing ischemic stroke through use of a combination of advanced genetic testing, risk stratification based on sociodemographic, dietary, and lifestyle factors is encouraged.

## Author contributions

SM: Conceptualization, Data curation, Funding acquisition, Methodology, Validation, Visualization, Writing – original draft, Writing – review & editing. AK: Supervision, Writing – original draft, Writing – review & editing.
